# Energy Harvesting and Task-Aware Multi-Robot Task Allocation in Robotic Wireless Sensor Networks

**DOI:** 10.3390/s23063284

**Published:** 2023-03-20

**Authors:** Omer Melih Gul

**Affiliations:** Department of Computer Engineering, Bahcesehir University, 34349 Istanbul, Turkey; omermelih.gul@bau.edu.tr

**Keywords:** multi-robot systems, task allocation, wireless networks, energy harvesting

## Abstract

In this work, we investigate an energy-aware multi-robot task-allocation (MRTA) problem in a cluster of the robot network that consists of a base station and several clusters of energy-harvesting (EH) robots. It is assumed that there are M+1 robots in the cluster and *M* tasks exist in each round. In the cluster, a robot is elected as the cluster head, which assigns one task to each robot in that round. Its responsibility (or task) is to collect the resultant data from the remaining *M* robots to aggregate and transmit directly to the BS. This paper aims to allocate the *M* tasks to the remaining *M* robots optimally or near optimally by considering the distance to be traveled by each node, the energy required for executing each task, the battery level at each node, and the energy-harvesting capabilities of the nodes. Then, this work presents three algorithms: Classical MRTA Approach, Task-aware MRTA Approach, EH and Task-aware MRTA Approach. The performances of the proposed MRTA algorithms are evaluated under both independent and identically distributed (i.i.d.) and Markovian energy-harvesting processes for different scenarios with five robots and 10 robots (with the same number of tasks). EH and Task-aware MRTA Approach shows the best performance among all MRTA approaches by keeping up to 100% more energy in the battery than the Classical MRTA Approach and keeping up to 20% more energy in the battery than the Task-aware MRTA Approach.

## 1. Introduction

### 1.1. Motivation

In 1999, *Business Week* considered wireless sensor networks (WSNs) as one of the 21 most important technologies for the 21st century [1]. The networking of smart and cheap small-size sensors over wireless links has many application areas, including video surveillance, traffic surveillance, air traffic control, physical security, military sensing, industrial and manufacturing automation, environment monitoring, structure monitoring, frost monitoring, health care, smart cities and distributed robotics [2,3].

WSNs are composed of many low-cost battery-powered wireless nodes which monitor their environment. Recently, wireless sensors and robot networks have emerged, adding mobile robots with rich resources to conventional WSNs. Robots can be used for performing automated tasks with no human intervention, even in a sparsely populated or hazardous environment. An application area of static sensor nodes might be volcanic areas where they can be used for measuring gas concentration, temperature, and other interesting values. In the case of detecting a dramatic change in parameters, an emergency situation named an ’event’ occurs. Events need to be analyzed via a robot equipped with more accurate sensing devices, such as seismometers, thermal cameras, etc. [4].

Recently, scholars have investigated the usage of robot deployment in numerous application areas, which include cleaning hazardous areas, harvesting fields, area exploration, battlefield surveillance, search and rescue missions, etc. Such complex domains decrease the possibility of single-robot usage since they are sensitive to failure, and task completion takes a long time. As they tend to be more fault-tolerant and robust, the demand for the solving of complex missions has driven multi-robot systems [5].

For the last two decades, robotics and WSNs have been well-investigated separately, so they are well-known fields. On the other hand, many new opportunities and research directions exist at the junction of these research fields, which are relatively unexplored. Until recently, robots and wireless sensors were considered separate network nodes. The survey [6] reviewed robotic applications in WSN. Robots and wireless sensors help each other in many aspects. Their combination, known as robotic and wireless sensor networks (RWSN), has several application areas such as military usage, transport monitoring, healthcare, weather forecasting, autonomous driving, mining, and search and rescue [7,8].

The multi-robot task-allocation (MRTA) problem is an important problem that needs to be solved efficiently for multi-robot systems to operate autonomously. The basic version of this problem (also known as the linear assignment problem) can be summarized as follows: Given a set of agents (or robots) and tasks, with each agent obtaining some payoff (or incurring some cost) for each task, find a one-to-one assignment of agents to tasks so that the overall payoff of all the agents is maximized (or cost is minimized) [9].

The MRTA problem may have many application areas including autonomous exploration [10], logistics [11], unmanned search and rescue missions [12], reconnaissance [13], etc. It is well known that the MRTA problem is NP-hard [14,15]. Therefore, it should be considered that proposed solutions have qualities inversely proportional to time complexity. A scenario given in [16] motivates our research and illustrates a correlation between industrial applications, multi-robot systems, and task allocation. Every hour, an online sales company sells a journal article. At the warehouse, a robot first receives an order. Second, it finds the corresponding item. Then, it prepares and packages items. Lastly, it sends it to the customer. What happens if the company is selling 20 articles each hour? Each minute? Each second?

The multi-robot systems which employ RF-domain security solutions may need computational and so energy resources if they use deep learning methods, as explained in [17,18,19,20]. Hence, multi-robot systems need more energy-efficient solutions for task execution.

To increase the network lifetime of a multi-robot system, energy efficiency should be considered. Therefore, this paper considers not only the traveled distance and battery level by each node but also the prediction of harvested energy at each node.

### 1.2. Our Contributions

The main contributions of this paper can be summarized as follows:

- To the best of our knowledge, this is the first work that considers the task-allocation problem with energy-harvesting nodes.
- When tackling the MRTA problem, our optimization includes all of the distance to be traveled by each node to reach its assigned task, the energy required for executing each task, the battery level at each node, and the energy-harvesting capabilities of the robot nodes.
- This work presents three MRTA algorithms: Classical MRTA Approach, Task-aware MRTA Approach, EH and Task-aware MRTA Approach.

### 1.3. Organization

The remainder of this paper is organized as follows. In Section 2, the related work is presented. We present the system model and define the problem in Section 3. In Section 4, we tackle the MRTA problem and propose three algorithms: Classical MRTA Approach, Task-aware MRTA Approach, EH and Task-aware MRTA Approach. In Section 5, we evaluate the performances of the proposed MRTA algorithms under different energy-harvesting processes for different scenarios with five robots and 10 robots (with same number of tasks). Section 6 concludes the paper and provides future research directions.

## 2. Related Work

This paper tackles the multi-robot task-allocation problem in energy-harvesting (EH) robot networks. Therefore, in this section, we first consider the related literature for the multi-robot task-allocation problem. Then, we consider the related literature for task-allocation problems in EH wireless sensor networks (WSN).

### 2.1. Multi-Robot Task Allocation

In recent years, there has been growing literature on the multi-robot task-allocation problem. In this section, we survey the recent papers in the MRTA literature.

The authors in [21] considered the simplest version of the multi-robot task-allocation (MRTA) problem in a multi-robot system and propose an optimal centralized solution, the Hungarian method. Despite its optimality, this kind of solution has the typical drawbacks of the centralized approach. For example, they show very slow responses to dynamic changes. Therefore, more distributed algorithms are proposed for this problem.

The authors in [22] considered an MRTA problem. An auction-based method is proposed for the task allocation to a group of robots. Tasks are considered to be some locations that the robots need to visit. A robot may be prevented from completing its allocated tasks using unexpected obstacles and delays. Therefore, the uncompleted tasks are rebid every time a robot completes its (previously) assigned task. This provides an opportunity to improve the allocation of the remaining tasks and to reduce the overall task-completion time.

The authors in [23] handled a MRTA problem in a multi-agent system. In this problem, there are tasks and identical agents where the number of tasks is less than the number of agents. Using distributed control laws, the agents are split into groups, each of which is assigned to a task. The paper suggests a distributed market-based solution. In the system, each agent has the information on all tasks and the maximum number of agents that can be assigned to each task. By considering the availability of the requested tasks, these agents communicate with each other to compare the bids and thus this knowledge propagates over the network.

The authors in [24] studied an initial formation problem in robotic swarm. Its goal is to minimize a certain objective function by determining which robot should go to each of the formation positions. The authors proposed an algorithm named Robot and Task Mean Allocation algorithm. In this algorithm, the cost is considered to be the difference between the distance from the robot to the task and the mean of distances from all the robots to that task. As a result, the robot will win the task that is best for the team, not only for itself.

In [25], a multi-robot task-allocation problem was studied in a distributed manner. It is assumed that each robot agent has the knowledge of its distance from the targets in the environment and the robots communicate with each other. A solution is found in a distributed manner without a shared memory of the system.

The authors in [26] studied an MRTA problem in robotic swarm. In this system, the number of robots is equal to the number of tasks and it is assumed that each robot can be assigned only one task. This paper proposed a distributed market-based MRTA algorithm whereby the robots are capable of bidding for tasks since the market-based approach provides a good trade-off between centralized and distributed algorithms.

In [27], the authors considered a dispatch problem in a multi-robot system. In each round, each robot is allowed to visit just one event, or several events in a sequence. The authors also considered the problem in a distributed manner with a wireless sensor network scenario where each event is sensed by a sensor and the sensor reports it to a robot. A pairwise distance-based matching algorithm is proposed to eliminate long matching edges by pairwise exchanges among two robot-event pairs to reduce overall path length. In [4], the authors extended the work [27] to a more general framework. They presented generalizations that handle multiple visits and timing constraints.

In [9], an offline task-assignment problem was studied in a distributed manner for a multi-robot system, where the tasks form disjoint groups and each robot has an upper bound on the number of tasks it can do both within the overall mission and within each task group. The aim was to find the best assignment of the robots to tasks so as to maximize the sum of the payoffs of all the robots. The authors propose centralized, decentralized, and distributed auction-based algorithms for the problem.

Luo et al. [28] studied the multi-robot task-assignment problem with task-deadline constraints. In this problem, the tasks form overlapping groups, and each robot can be assigned multiple tasks in each group.

The work in [29] formulated a single-robot task, a single-task robot, time-extended assignment, and MRTA problem with multiple, nonlinear criteria using discrete variables that drastically reduce the computation burden. The proposed Branch and Bound (B&B) technique tackles low-scale MRTA problems while the proposed genetic algorithm (GA) specifically considers large-scale MRTA problems. The proposed MRTA techniques consider three optimization criteria simultaneously: traveled distance, task execution time, and energetic feasibility. Although the work considers thermosolar power plants as a case study, the proposed algorithms can be used for any multi-criteria MRTA problem with a nonlinear cost function equivalently. Performance and response of proposed methods are evaluated in various scenarios. It is shown that the B&B technique can reach global optimality within a reasonable time. GA converges global optimality within less computational time for larger problems. Furthermore, a trade-off between accuracy and computation time can be carried out easily via parameter tuning by considering the computational power.

The work in [30] considers an in-schedule-dependent MRTA problem. A main issue with those problems is that combinatorial optimization is inherently NP-hard. This work presents a decentralized MRTA technique by leveraging submodularity and a sampling process of tasks. It is revealed analytically that within polynomial time complexity, it can guarantee approximately half of the optimal solutions for monotone submodular case and a quarter for the nonmonotone submodular case. To investigate its performance and validate theoretical analysis, two MRTA scenarios are introduced for numerical simulations. They show that it achieves a solution quality comparable to the state-of-the-art algorithms in the monotone case and better quality in the nonmonotone case, with considerably less computation complexity.

### 2.2. Task Allocation in Energy-Harvesting (EH) Wireless Sensor Networks (WSN)

Recently, many works have studied task-allocation problems in EH-WSN [31,32,33]. In the literature on task allocation in WSN [31,32,33], based on the known static energy models, the tasks are assigned to sensor nodes given initially available resources and a directed acyclic graph (DAG). However, the time-varying nature of EH-WSNs brings new considerations for energy models and the design of task-allocation schemes.

Many works in the literature tackle resource management for real-time energy-harvesting embedded systems. In [34,35], dynamic voltage scaling policies decrease energy consumption. Nevertheless, they may violate the scheduling length constraint for task allocation to sensors. The works in [36,37] present energy-clairvoyant lazy scheduling algorithms. Lazy scheduling algorithms can be categorized as non-work-conserving scheduling disciplines where a lazy scheduler may be idle although waiting tasks are ready to be processed. In addition, the work assumes that tasks are preemptive and independent of each other. More precisely, the currently active task may be preempted at any time and have its execution resumed later, at no additional cost. Additionally, since LSA is only based on an “as late as possible” heuristic, it is more likely that the battery overflows due to harvested energy, which results in missed-recharging opportunities. Moreover, the abovementioned works mainly focus on the task allocation to a single processor with an energy-harvesting capability but not on the allocation of a task graph with precedence constraints to multiple nodes.

The most closely related works are [38,39], and are closely related to our paper. Unlike conventional computing-oriented scheduling methods, the paper [38] introduces a dynamic energy-oriented scheduling algorithm. By conducting decomposition, combination, concurrent execution, and admission control, their proposed method allocates tasks based on the dynamically changed available energy. Evaluation results with indoor and outdoor settings show that the proposed technique has very little energy consumption overhead, so it is extremely lightweight. It schedules tasks effectively using dynamically available energy. The work in [39] tackled the task-allocation problem for EH-WSNs. The aim was to maximize the network profit while ensuring the task executions within given WSN lifetimes. It provided an analytical approach. Then, it presented a distributed mission assignment scheme for wireless sensor nodes with rechargeable batteries.

The work in [40] tackled the EH-aware task-allocation problem at the network level. In other words, it considered assigning nodes to the tasks within precedence constraints from the task graph. This differed this problem from the previous task-allocation problems focusing on scheduling individual tasks at node level. Second, it aimed for network reward maximization over the time horizon instead of facilitating network operation perennially.

## 3. System Model and Problem Definition

This paper tackles a multi-robot task-allocation problem in EH robot networks. In this section, we will present a motivating scenario and formulate the problem based on this motivation. We interchangeably use robot, node, and sensor in the rest of this paper. First, we consider the system model of the robotic network. Then, we define the task-allocation problem more precisely.

### 3.1. System Model

**Figure 1:**
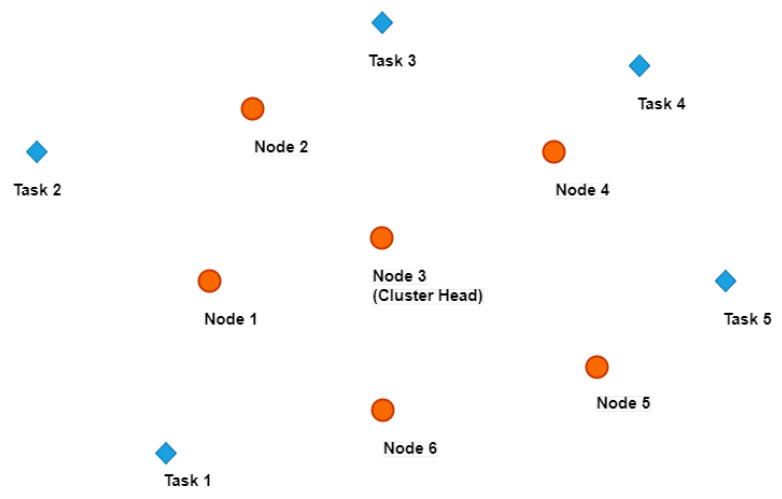
An example of a networked system consists of six robot nodes. Here, robot node 3 is the cluster head robot that assigns one task to each of the remaining five robots. When executing the tasks, these robots benefit from the data collected from the sensors. On the other hand, these sensors are removed for simplicity purposes while illustrating the MRTA problem.

In this work, we consider a cluster in a robot network that consists of a base station (BS) and several clusters of mobile robotic sensors. Please see Figure 1 as an example of the multi-robot system. The index set of all robots in the cluster is denoted by *S*. This cluster is composed of M+1 robots and *M* tasks emerge in each round *t*. In a cluster, one of the robots is elected as the cluster head. The cluster head in round *t* is denoted by H(t) and H(t)=i if the robot *i* is the cluster head in round *t*. In the multi-robot system, the cluster head robot collects data from the remaining *M* robots to aggregate and send directly to the BS. $B_{i}\left(t\right)$ is the energy remaining in the battery of robot *i* in TS *t* and $E_{i}^{h}\left(t\right)$ is the energy harvested by robot *i* in TS *t*.

### 3.2. Problem Definition

We consider the energy-aware multi-robot task-allocation problem as a matching dispatch problem. (One robot to one task assignment is a matching dispatch problem). A *M*-to-*M* task allocation (or matching) algorithm is defined as follows.

**Definition** **1.** *An M-to-M task-allocation algorithm, denoted by π, is a one-to-one matching function that assigns one task j to a robot i in round t. This matching function for round t is denoted by π(·,t):i→j where i∈S−{H(t)} and j∈{1,…,M}.*

Unlike most task-allocation algorithms for matching dispatch problems, we also consider the remaining energy in the robot batteries in the multi-robot task-allocation problem. We should not assign a task to a robot if the robot does not have enough energy to complete that task after reaching the position of that task (sometimes “event” or “target” is used).

$E(T_{i},t)$ denotes the energy consumed by node *i* to execute task $T_{i}\left(t\right)$ in round *t*.

**Definition** **2.** *Residual energy in round t, denoted by $B_{i}\left(t\right)$, is the energy remaining in the robot’s battery i when it reaches the position of the assigned task, i.e.,*

(1) $$B_{i}\left(t+1\right)≜B_{i}\left(t\right)+\sum_{\tau =1}^{T}E_{i}^{h}\left(\tau \right)-∥\xi_{i}\left(t\right)-\xi_{\pi }\left(i,t\right)∥E_{pc}-E\left(T_{i},t\right)$$

*where $E_{pc}$ is a constant energy for one unit position change in a robot; $\xi_{i}\left(t\right)$ and $\xi_{\pi }\left(i,t\right)$ denote the position of robot i and the position of the task assigned to robot i in round t under the algorithm π, respectively.*

Based on these factors, we define a quadratic cost for a robot and its assigned task. As well as this, we add a constraint (a Kuhn–Tucker condition) to prevent any robot from suffering from a lack of energy (becoming a dead node) and not completing its assigned task. From Definition 1 and Definition 2, the multi-robot task-allocation (MRTA) problem is defined as a constrained optimization problem [41] as follows.

**Problem** **1.** *Energy-harvesting-aware multi-robot task-allocation problem*

$$\begin{array}{ccc} \; & \underset{\pi \in G}{min}\; & \;\;\;\;\;\;\sum_{s_{i}\in S-\left\{H\left(t\right)\right\}}\;\;\left[{\left(B_{i}\left(t\right)+\sum_{\tau =1}^{K}E_{i}^{h}\left(\tau \right)-∥\xi_{i}\left(t\right)-\xi_{\pi }\left(i,t\right)∥\times E_{pc}-E\left(T_{i},t\right)\right)}^{2}+\left(\lambda_{a}-B_{i}\left(t\right)\right)\right]\\ \; & s.t.\; & \lambda_{a}<B_{i}\left(t\right) \end{array}$$

where *G* is the set of all matching dispatch algorithms completing; $\lambda_{a}$ is the required energy for a node to stay alive (to avoid becoming a dead node); and *K* is the number of time slots in each round.

Table 1 provides the notations and their explanations for ease of reference.

## 4. Proposed Multi-Robot Task-Allocation (MRTA) Approaches

In this section, we tackle the MRTA problem (in Problem 1) which includes the distance to be traveled by each node, the energy required for executing each task, the battery level at each node and the energy-harvesting capabilities of the nodes.

By considering different subsets of these parameters, we present Classical MRTA Approach, Task-aware MRTA Approach, EH and Task-aware MRTA Approach.

### 4.1. Classical MRTA Approach

In this subsection, we tackle the MRTA problem (in Problem 1) by considering the distance to be traveled by each node and the battery level at each node.

We propose a solution based on the Hungarian algorithm [21]. Please note that for *M* number of robots, the computational complexity of the optimal Hungarian algorithm is $O(M^{3})$. Our proposed solution in this section does not consider the energy-harvesting capabilities of the nodes.

Our proposed solution is given in Algorithm 1.
**Algorithm 1** **Classical MRTA Algorithm****Initialization:** The index set of tasks is $T=\{T_{1},\ldots ,T_{M}\}$.
 **Algorithm:**

 **(1)** Match each robot with a task.
 **for** i=1:M
  **(2)** For each robot *i*, calculate the costs *all of the traveled distance and battery level by each node*.
  **(3)** For each robot *i*, look at the costs of other robots.
  **(4)** If the cost decreases when the two robots change the tasks each other.
  **(5)** The robots swap their assigned tasks.
  **(6)** Otherwise, robots do not change their assigned tasks.
 **end**


The next subsection presents the Task-aware MRTA Approach, which considers the energy required for executing each task different from the Classical MRTA Approach.

### 4.2. Task-Aware MRTA Approach

In this subsection, we tackle the MRTA problem (in Problem 1) by considering the distance to be traveled by each node, the energy required for executing each task, and the battery level at each node.

We propose the Task-aware MRTA Approach based on the Hungarian algorithm [21]. Please notice that for *M* number of robots, the computational complexity of the optimal Hungarian algorithm is $O(M^{3})$. Our proposed solution in this section does not consider the energy-harvesting capabilities of the nodes.

Our proposed solution is given in Algorithm 2.
**Algorithm 2** **Task-aware MRTA Algorithm****Initialization:** The index set of tasks is $T=\{T_{1},\ldots ,T_{M}\}$.
 **Algorithm:**
 **(1)** Match each robot with a task.
 **for** i=1:M
  **(2)** For each robot *i*, calculate the costs *all of the traveled distance, battery level by each node and the energy required for executing each task*.
  **(3)** For each robot *i*, look at the costs of other robots.
  **(4)** If the cost decreases when the two robots change the tasks each other.
  **(5)** The robots swap their assigned tasks.
  **(6)** Otherwise, robots do not change their assigned tasks.
 **end**


### 4.3. EH and Task-Aware MRTA Approach

In this section, we tackle the MRTA problem (in Problem 1) by considering all the traveled distance and battery level by each node, the energy required for executing each task and the prediction of harvested energy at each node. We can predict the energy-harvesting capabilities of the nodes.

We propose the EH and Task-aware MRTA Approach based on the Hungarian algorithm [21]. Please notice that for *M* number of robots, the computational complexity of the optimal Hungarian algorithm is $O(M^{3})$.

Our proposed solution is given in Algorithm 3.
**Algorithm 3** **EH and Task-aware MRTA Algorithm****Initialization:** The index set of tasks is $T=\{T_{1},\ldots ,T_{M}\}$.
 **Algorithm:**
 **(1)** Match each robot with a task.
 **for** i=1:M
  **(2)** For each robot *i*, calculate the costs *all of the traveled distance and battery level by each node, the energy required for executing each task and the prediction of harvested energy at each node*.
  **(3)** For each robot *i*, look at the costs of other robots.
  **(4)** If the cost decreases when the two robots change the tasks with each other.
  **(5)** The robots swap their assigned tasks.
  **(6)** Otherwise, robots do not change their assigned tasks.
 **end**


Many algorithms have recently been proposed to predict energy harvesting processes [42]. As a robust solution, we use a weighted moving average-based algorithm for predicting energy-harvesting processes.

## 5. Numerical Results

As stated in Section 2, the pioneering approach for the MRTA problem is the Hungarian algorithm [21]. As we consider the MRTA problem by also considering the energy-harvesting capabilities of nodes, the problem is changed. Therefore, this paper compares the proposed EH and Task-aware MRTA Approach with two benchmark policies—the Classical MRTA algorithm, and the Task-aware MRTA algorithm, which are based on the Hungarian algorithm [21].

In this section, we evaluate the performance of the proposed algorithms (Classical MRTA Approach, Task-aware MRTA Approach, EH and Task-aware MRTA Approach) under both independent and identically distributed (i.i.d.) and Markov EH processes. For each process, we consider two cases: five robot–five task and 10 robot–10 task cases.

Normally, the number of robots is one more than the number of tasks, because the cluster head robot does not execute a task, but just assigns one task to one robot in each round. To focus on the one-to-one matching problem, we ignore the location of the cluster head robot in the figures in this section.

Numerical experiments are conducted on a 1000 m × 1000 m field where a robot can move to the location of its assigned task with a velocity of *v* = 10 m/s. At the beginning, each node has a full battery that can store $B_{i}$ = 600 mJ energy.

### 5.1. Independent and Identically Distributed (I.I.D.) EH Process

In this subsection, we will consider two cases under i.i.d. EH process: five robot–five task task and 10 robot–10 task cases. The i.i.d. process at each node is generated as a Poisson process with different means for each node.

#### 5.1.1. 5 I.I.D. EH Robot Case

In this subsubsection, we will consider a case with five i.i.d. EH robots and five tasks.

**Figure 2:**
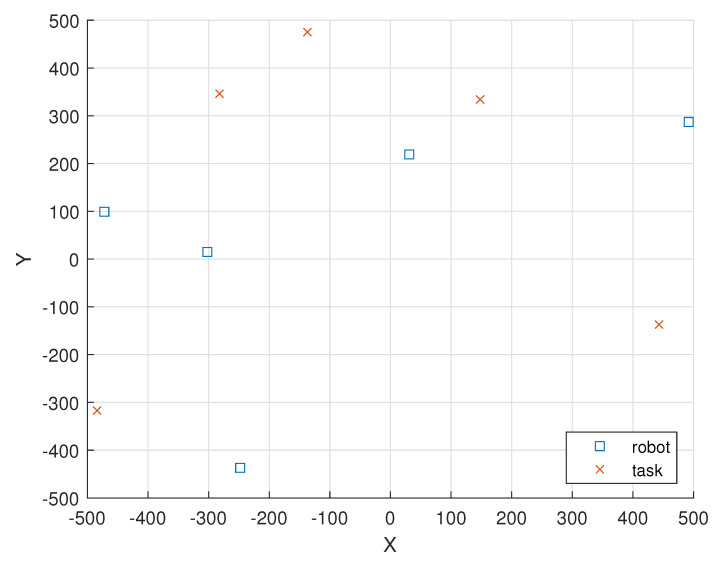
The locations of the 5 i.i.d. EH robot nodes and the locations of the five tasks are represented as “square” and “cross” markers, respectively.

Figure 2 illustrates the locations of the five i.i.d. EH robot nodes and the locations of the five tasks in a 1000 m × 1000 m field.

In Figure 2, the locations of the five i.i.d. EH robot nodes are given as $(\xi_{1}\left(0\right),\xi_{2}\left(0\right),\xi_{3}\left(0\right)$,

$\xi_{4}\left(0\right),\xi_{5}\left(0\right))=\left(\left(-472,99\right),\left(-302,15\right),\left(31,219\right),\left(492,287\right),\left(-248,-437\right)\right)$.

In Figure 2, the locations of the five tasks in round 1 are given as (ξ(1,1),ξ(2,1),ξ(3,1),

ξ(4,1),ξ(5,1))=((−282,346),(−137,475),(148,334),(443,−137),(−484,−317)).

**Figure 3:**
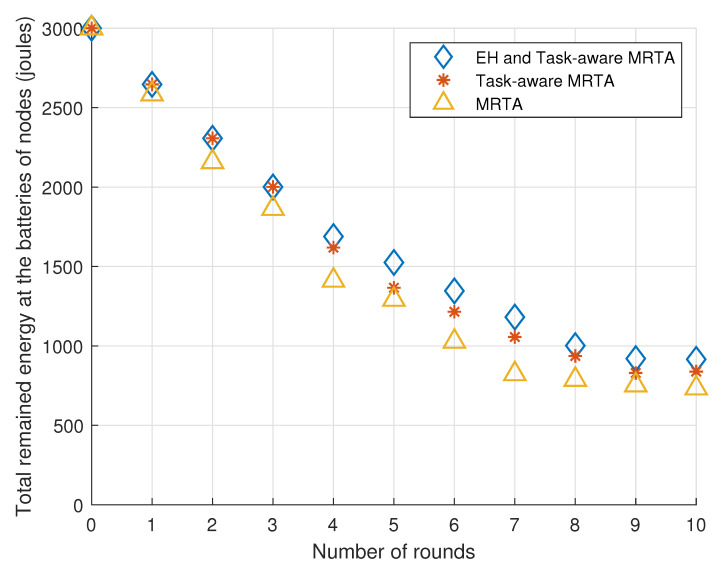
Total remaining energy in the batteries of the 5 i.i.d. EH robot nodes versus the number of rounds under i.i.d. EH processes.

Figure 3 exhibits the total remaining energy in the batteries of the five i.i.d. EH robot nodes versus the number of rounds under Poisson i.i.d. EH processes.

Considering the general trend, EH and Task-aware MRTA and Task-aware MRTA show better performance than MRTA. This is expected because Task-aware MRTA considers the energy to be consumed for each task execution, while conventional MRTA does not consider this energy. In addition to energy to be consumed for each task execution, EH and Task-aware MRTA consider the energy-harvesting process at each node by predicting harvested energy. Therefore, EH and Task-aware MRTA shows better performance than not only conventional MRTA but also Task-aware MRTA.

Table 2 shows the remaining total energy in the batteries of the five i.i.d. EH robot nodes, which are located initially as given in Figure 2 versus the number of rounds under i.i.d. EH processes. As there are five robots, the total remaining energy in the batteries of the nodes is 5×600=3000 mJ initially (denoted as round 0).

From Table 2, we can make the following observations. In the first three rounds, EH and Task-aware MRTA performs the same as Task-aware MRTA, where both algorithms shows slightly better performance (stores 7.5%-more energy) than (conventional) MRTA algorithm. In round 4, EH and Task-aware MRTA start to show better performance than Task-aware MRTA, too. Especially in round 7, EH and Task-aware MRTA shows its best relative performance (43.6% more) compared with MRTA. Task-aware MRTA also shows its best relative performance (28.3% more) compared with MRTA. In addition, EH and Task-aware MRTA shows its best relative performance (11.9% more) compared with Task-aware MRTA in round 7.

#### 5.1.2. 10 I.I.D. EH Robot Case

In this subsubsection, we will consider a case with 10 i.i.d. EH robots and 10 tasks.

**Figure 4:**
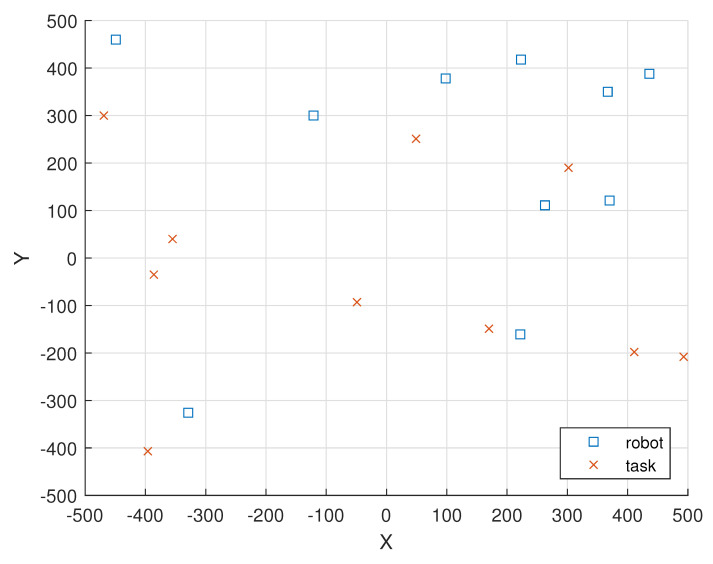
The locations of the 10 i.i.d. EH robot nodes and the locations of the 10 tasks are represented as “square” and “cross” markers, respectively.

Figure 4 illustrates the locations of the 10 i.i.d. EH robot nodes and the locations of the 10 tasks in a 1000 m × 1000 m field.

In Figure 4, the locations of the 10 i.i.d. EH robot nodes are given as $(\xi_{1}\left(0\right),\xi_{2}\left(0\right),\xi_{3}\left(0\right)$,

$\xi_{4}\left(0\right),\xi_{5}\left(0\right),\xi_{6}\left(0\right),\xi_{7}\left(0\right),\xi_{8}\left(0\right),\xi_{9}\left(0\right),\xi_{10}\left(0\right))=\left(367,350\right),\left(263,111\right),\left(436,388\right),\left(223,418\right)$,

(−121,300),(222,−161),(98,378),(−449,460),(−329,−326),(370,121)).

In Figure 4, the locations of the 10 tasks in round 1 are given as (ξ(1,1),ξ(2,1),ξ(3,1),

ξ(4,1),ξ(5,1),ξ(6,1),ξ(7,1),ξ(8,1),ξ(9,1),ξ(10,1))=((−469,300),(−386,−35),(−355,40),(−49,−93),(49,251),(302,190),(493,−208),(411,−198),(170,−149),(−396,−407)).

**Figure 5:**
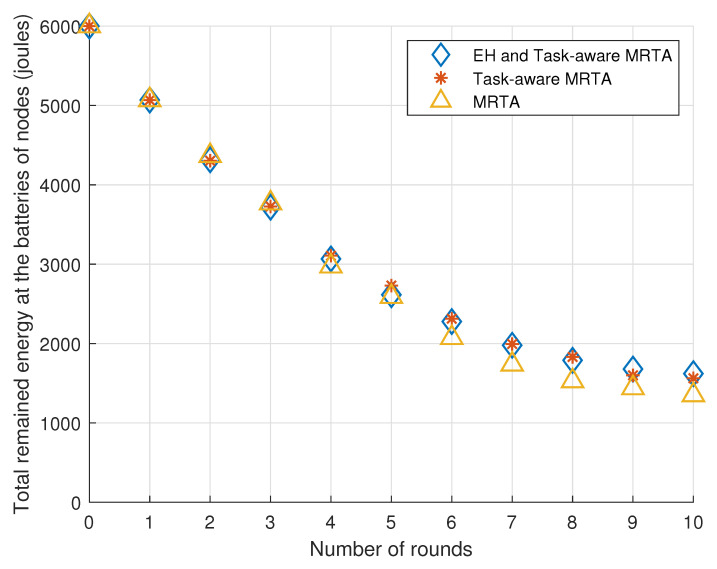
Total remaining energy in the batteries of the 10 robot nodes versus the number of rounds under i.i.d. EH processes.

Figure 5 exhibits the total remaining energy in the batteries of the 10 i.i.d. EH robot nodes versus the number of rounds under Poisson i.i.d. EH processes.

Considering the general trend, EH and Task-aware MRTA and Task-aware MRTA show better performance than MRTA. This is expected because Task-aware MRTA considers the energy to be consumed for each task execution while conventional MRTA do not consider this energy. In addition to energy to be consumed for each task execution, EH and Task-aware MRTA consider the energy-harvesting process at each node by predicting harvested energy. Therefore, EH and Task-aware MRTA shows better performance than not only conventional MRTA but also Task-aware MRTA.

Table 3 shows the remaining total energy in the batteries of the 10 robot nodes located initially as given in Figure 4 versus the number of rounds under Poisson EH processes. As there are 10 robots, the total remaining energy in the batteries of the nodes is 10 × 600 = 6000 mJ initially (denoted as round 0).

From Table 3, we can make the following observations. In the first five rounds, EH and Task-aware MRTA performs the same as Task-aware MRTA where both algorithms show slightly better performance (stores at most 10% more energy) than the (conventional) MRTA algorithm. In later rounds, EH and Task-aware MRTA starts to show better performance than Task-aware MRTA and MRTA. In round 10, EH and Task-aware MRTA shows its best relative performance (20% more) compared with MRTA. Task-aware MRTA also shows its best relative performance (17% more) compared with MRTA in round 8.

### 5.2. Markov EH Process

In this subsection, we will consider two cases under the Markov EH process: five robot–five task and 10 robot–10 task cases. The Markov process at each node is generated as a Markov process with a different mean for each node.

#### 5.2.1. 5 Markov EH Robot Case

This subsubsection will consider a case with five Markov EH robots and five tasks.

**Figure 6:**
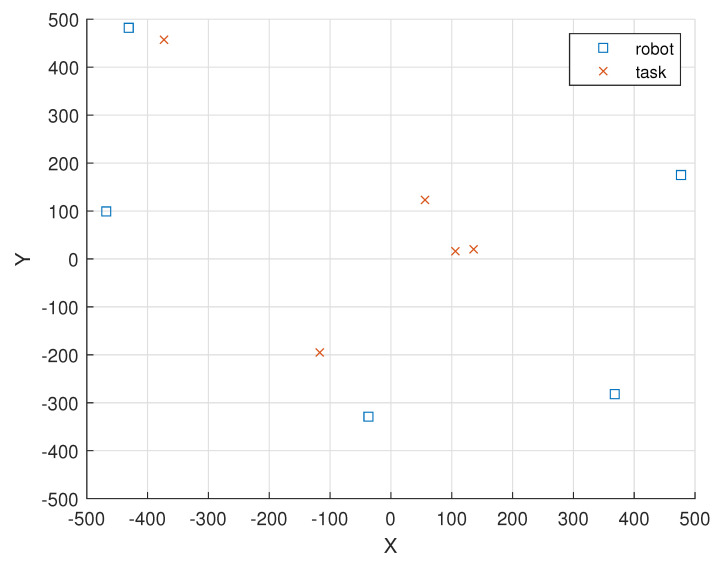
The locations of the five Markov EH robot nodes and the locations of the five tasks are represented as “square” and “cross” markers, respectively.

Figure 6 illustrates the locations of the five Markov EH robot nodes and the locations of the five tasks in a 1000 m × 1000 m field.

In Figure 6, the locations of the five Markov EH robot nodes are given as $(\xi_{1}\left(0\right),\xi_{2}\left(0\right)$,

$\xi_{3}\left(0\right),\xi_{4}\left(0\right),\xi_{5}\left(0\right))=\left(\left(477,175\right),\left(368,-282\right),\left(-468,99\right),\left(-37,-329\right),\left(-431,482\right)\right)$.

In Figure 6, the locations of the five tasks in round 1 are given as (ξ(1,1),ξ(2,1),ξ(3,1),

ξ(4,1),ξ(5,1))=((−373,457),(56,123),(106,16),(136,20),(−117,−195)).

**Figure 7:**
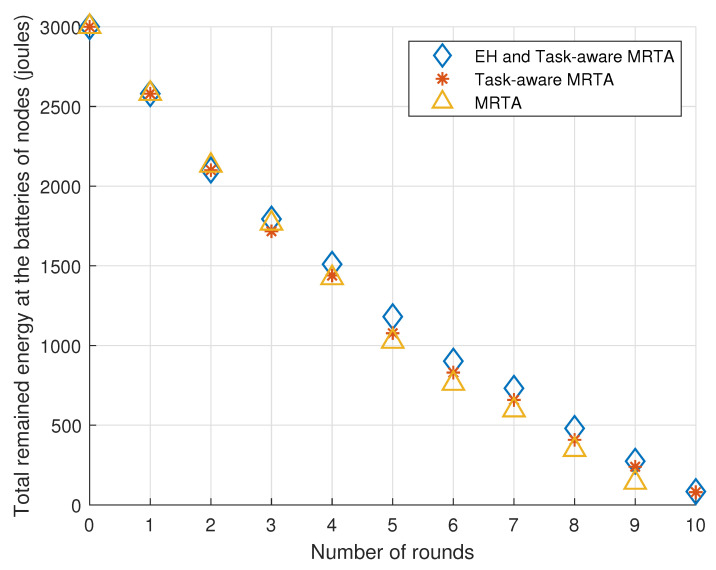
Total remaining energy in the batteries of the five Markov EH robot nodes versus the number of rounds under Markov EH processes.

Figure 7 exhibits the total remaining energy in the batteries of the five Markov EH robot nodes versus the number of rounds under Markov EH processes.

Considering the general trend, EH and Task-aware MRTA and Task-aware MRTA show better performance than MRTA. This is expected because Task-aware MRTA considers the energy to be consumed for each task execution while conventional MRTA does not consider this energy. In addition to energy to be consumed for each task execution, EH and Task-aware MRTA considers the energy-harvesting process at each node by predicting harvested energy. Therefore, EH and Task-aware MRTA shows better performance than both conventional MRTA and Task-aware MRTA. It should be noted that in round 10, conventional MRTA cannot even do task execution due to insufficient stored energy in the batteries of the nodes. As Markov EH process has a different memory from the i.i.d. EH process, its prediction is harder. As a result, the total energy stored under Markov EH process is less than i.i.d. EH process.

Table 4 shows the remaining total energy in the batteries of the five Markov EH robot nodes located initially as given in Figure 6 versus the number of rounds under Markov EH processes. As there are five robots, the total remaining energy in the batteries of the nodes is 5×600=3000 mJ initially (denoted as round 0).

From Table 4, we can make the following observations. In first four rounds, EH and Task-aware MRTA performs same as Task-aware MRTA where both algorithms show slightly better performance (stores at most 6% more energy) than the (conventional) MRTA algorithm. In round 9, EH and Task-aware MRTA start to show much better performance than Task-aware MRTA and MRTA, so that EH and Task-aware MRTA stores twice more energy as MRTA. Moreover, EH and Task-aware MRTA shows its best relative performance (14.65% more) compared with Task-aware MRTA in round 9. In round 10, EH and Task-aware MRTA and Task-aware MRTA cannot show relative performance because MRTA even cannot show any performance due to a lack of energy.

#### 5.2.2. 10 Markov EH Robot Case

In this subsubsection, we will consider a case with 10 Markov EH robots and 10 tasks.

**Figure 8:**
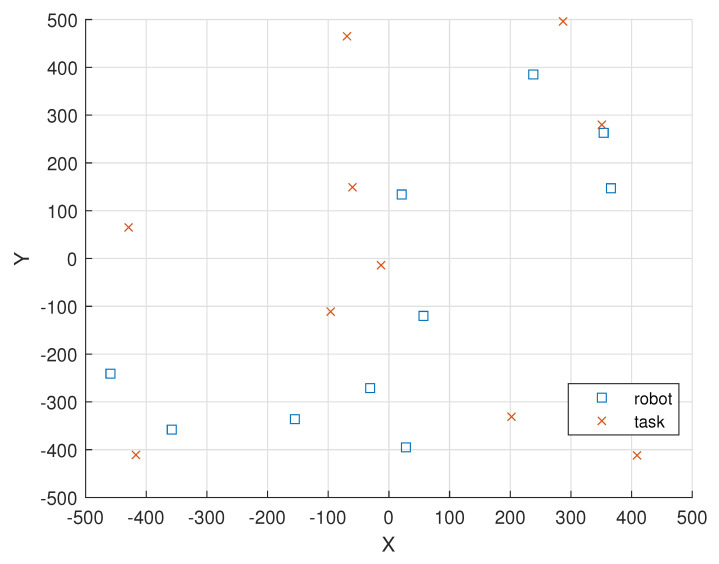
The locations of the 10 Markov EH robot nodes and the locations of the 10 tasks are represented as “square” and “cross” markers, respectively.

Figure 8 illustrates the locations of the 10 Markov EH robot nodes and the locations of the 10 tasks in a 1000 m × 1000 m field.

In Figure 8, the locations of the 10 robot nodes are given as $(\xi_{1}\left(0\right),\xi_{2}\left(0\right),\xi_{3}\left(0\right),\xi_{4}\left(0\right)$,

$\xi_{5}\left(0\right),\xi_{6}\left(0\right),\xi_{7}\left(0\right),\xi_{8}\left(0\right),\xi_{9}\left(0\right),\xi_{10}\left(0\right))=\left(-31,-271\right),\left(366,147\right),\left(21,134\right),\left(-155,-336\right)$,

(−358,−358),(−459,−241),(57,−120),(28,−395),(238,385),(354,263)).

In Figure 8, the locations of the 10 tasks in round 1 are given as (ξ(1,1),ξ(2,1),ξ(3,1),

ξ(4,1),ξ(5,1),ξ(6,1),ξ(7,1),ξ(8,1),ξ(9,1),ξ(10,1))=((−429,65),(−60,149),(−69,465),

(287,496),(351,280),(409,−412),(202,−331),(−13,−14),(−96,−111),(−417,−411)).

**Figure 9:**
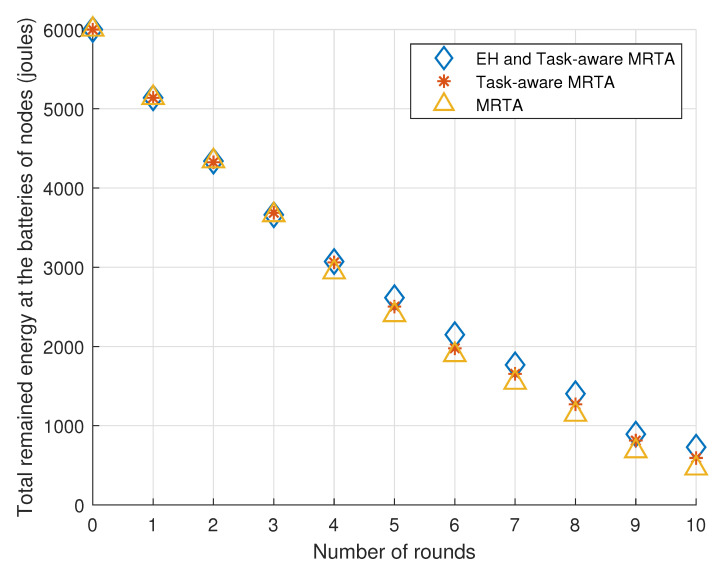
Total remaining energy in the batteries of the 10 robot nodes versus the number of rounds under Markov EH processes.

Figure 9 exhibits the total remaining energy in the batteries of the 10 Markov EH robot nodes versus the number of rounds under Markov EH processes.

Considering the general trend, EH and Task-aware MRTA and Task-aware MRTA show better performance than MRTA. This is expected because Task-aware MRTA considers the energy to be consumed for each task execution while conventional MRTA does not consider this energy. In addition to energy to be consumed for each task execution, EH and Task-aware MRTA considers the energy-harvesting process at each node by predicting harvested energy. Therefore, EH and Task-aware MRTA shows better performance than not only conventional MRTA but also Task-aware MRTA. As the Markov EH process has a memory different from i.i.d. EH process, its prediction is harder. The Markov process has a memory, so the energy harvests under the Markov process are more correlated than those under the i.i.d. process. The energy harvested under the Markov process is considerably lower than that under the i.i.d. process. As a result, the total energy stored under the Markov EH process is less than the i.i.d. EH process.

Table 5 shows the remaining total energy in the batteries of the 10 Markov EH robot nodes, which are located initially as given in Figure 8 versus the number of rounds under Markov EH processes. As there are 10 robots, the total remaining energy in the batteries of the nodes is 10×600=6000 mJ initially (denoted as round 0).

From Table 5, we can make the following observations. In the first five rounds, EH and Task-aware MRTA performs the same as Task-aware MRTA, where both algorithms show slightly better performance (stores at most 9.1% more energy) than the (conventional) MRTA algorithm. In round 8, EH and Task-aware MRTA start to show much better performance than Task-aware MRTA and MRTA. Especially in round 10, EH and Task-aware MRTA shows its best relative performance (57.5%) compared with MRTA in round 10. Task-aware MRTA also shows its best relative performance (28.1% more) compared with MRTA in round 10. Moreover, EH and Task-aware MRTA shows its best relative performance (23.0%) compared with Task-aware MRTA in round 10.

## 6. Conclusions

In this work, we investigate a multi-robot task-allocation (MRTA) problem occurring in a cluster of robots. In the cluster, a robot operates as a cluster head. Each of the remaining (non-cluster head) robots is assigned to do a task in a round. This task-allocation algorithm between robots and tasks considers both distances and remaining energy while assigning tasks to the robots. The problem is defined as a one-to-one matching dispatch problem.

This paper tackles the multi-robot task-allocation (MRTA) problem by considering the distance to be traveled by each node, the energy required for executing each task, the battery level at each node, and the energy-harvesting capabilities of the nodes. Then, this work presents three algorithms: Classical MRTA Approach, Task-aware MRTA Approach, and EH and Task-aware MRTA Approach. The performances of the proposed MRTA algorithms are evaluated for different energy-harvesting processes and the different number of robots (with the same number of tasks).

In contrast to classical MRTA and Task-aware MRTA Approaches, EH and Task-aware MRTA Approach considers all of the distance to be traveled by each node, the energy required for executing each task, the battery level at each node, and the energy-harvesting capabilities of the nodes. Therefore, it shows the best performance among all MRTA approaches by keeping up to 100% more energy in the battery than the Classical MRTA Approach and keeping up to 20% more energy in the battery than the Task-aware MRTA Approach.

In the future, we will consider low-complexity MRTA approaches in the related literature other than the Hungarian algorithm. Thus, we aim to propose a more robust energy-aware multi-robot task-allocation (MRTA) algorithm in energy-harvesting wireless sensor networks.

## Figures and Tables

**Table 1:** Notations and Their Explanations.

Notation	Explanations
*S*	Index set of robots
*M*	Number of tasks
M+1	Number of robots
*t*	Time slot
*K*	The number of time slots in each round
H(t)	The cluster head in round *t*
$B_{i}\left(t\right)$	Remained energy in the battery of *i*th robot
$E_{i}^{h}\left(t\right)$	Energy harvested by robot *i* in TS *t*
π	*M*-to-*M* task-allocation algorithm
*G*	Set of all matching dispatch algorithms
*T*	Index set of tasks
$T_{i}$	*i*th task
$\xi_{i}\left(t\right)$	Position of robot *i*
$\xi_{\pi }\left(i,t\right)$	Position of the task assigned to robot *i* in round *t* under the algorithm π
$E(T_{i},t)$	Energy consumed by node *i* to execute task $T_{i}\left(t\right)$ in round *t*
$E_{pc}$	Energy consumed by a node to travel unit distance (position change)

**Table 2:** Total remaining energy in the batteries of the five robot nodes which are located initially as given in Figure 2 versus the number of rounds under i.i.d. EH processes.

Round	0	1	2	3	4	5	6	7	8	9	10
EH & Task-aware MRTA	3000	2646	2307	2001	1689	1525	1346	1182	1002	920	916
Task-aware MRTA	3000	2646	2307	2001	1619	1366	1215	1056	937	829	838
MRTA	3000	2584	2158	1864	1412	1291	1027	823	788	753	733
EH & Task-aware MRTA/MRTA	1.000	1.024	1.069	1.073	1.196	1.181	1.311	1.436	1.272	1.222	1.250
Task-aware MRTA/MRTA	1.000	1.024	1.069	1.073	1.147	1.058	1.183	1.283	1.189	1.101	1.143

**Table 3:** The total remaining energy in the batteries of the 10 robot nodes, which are located initially as given in Figure 4 versus the number of rounds under Poisson EH processes.

Round	0	1	2	3	4	5	6	7	8	9	10
EH & Task-aware MRTA	6000	5069	4365	3768	3107	2615	2313	1994	1830	1678	1620
Task-aware MRTA	6000	5069	4316	3727	3067	2731	2277	1978	1789	1600	1563
MRTA	6000	5069	4304	3718	2973	2589	2072	1737	1526	1439	1351
EH & Task-aware MRTA/MRTA	1.000	1.000	1.014	1.013	1.045	1.110	1.116	1.148	1.199	1.166	1.199
Task-aware MRTA/MRTA	1.000	1.000	1.003	1.002	1.032	1.055	1.099	1.139	1.172	1.112	1.157

**Table 4:** Total remaining energy in the batteries of the five Markov EH robot nodes, which are located initially as given in Figure 6 versus the number of rounds under Markov EH processes. “NA” denotes not applicable.

Round	0	1	2	3	4	5	6	7	8	9	10
EH & Task-aware MRTA	3000	2580	2101	1794	1510	1181	902	732	480	274	84
Task-aware MRTA	3000	2580	2101	1766	1439	1077	830	659	408	239	80
MRTA	3000	2580	2129	1717	1424	1025	761	594	346	140	NA
EH & Task-aware MRTA/MRTA	1.000	1.000	1.013	1.042	1.060	1.152	1.185	1.232	1.179	1.957	NA
Task-aware MRTA/MRTA	1.000	1.000	1.013	1.029	1.011	1.051	1.091	1.109	1.179	1.707	NA

**Table 5:** Total remaining energy in the batteries of the 10 robot nodes which are located initially as given in Figure 8 versus the number of rounds under Markov EH processes.

Round	0	1	2	3	4	5	6	7	8	9	10
EH & Task-aware MRTA	6000	5138	4339	3662	3072	2616	2149	1768	1404	894	729
Task-aware MRTA	6000	5138	4325	3687	3061	2503	1977	1655	1270	813	593
MRTA	6000	5138	4339	3659	2937	2398	1893	1544	1140	680	463
EH & Task-aware MRTA/MRTA	1.000	1.000	1.000	1.016	1.046	1.091	1.135	1.145	1.232	1.315	1.575
Task-aware MRTA/MRTA	1.000	1.000	0.997	1.001	1.042	1.044	1.044	1.072	1.114	1.196	1.281

## Data Availability

Not applicable.

## Abbreviations

The following abbreviations are used in this manuscript:

MRTA	Multi-robot Task Allocation
EH	Energy Harvesting
WSN	Wireless Sensor Network
RWSN	Robotic and Wireless Sensor Network
EH-WSN	Energy-Harvesting Wireless Sensor Network
IID	Independent and identically distributed
GA	Genetic Algorithm
B&B	Branch and Bound Algorithm

## References

[1] (1999). 21 ideas for the 21st century. Business Week.

[2] Chong C.-Y., Kumar S.P. (2003). Sensor networks: Evolution, opportunities, and challenges. Proc. IEEE.

[3] Akyildiz I.F., Su W., Sankarasubramaniam Y., Cayirci E. (2002). A survey on sensor networks. IEEE Commun. Mag..

[4] Lukic M., Barnawi A., Stojmenovic I. (2015). Robot Coordination for Energy-Balanced Matching and Sequence Dispatch of Robots to Events. IEEE Trans. Comput..

[5] Gautam A., Thakur A., Dhanania G., Mohan S. A distributed algorithm for balanced multi-robot task allocation. Proceedings of the 2016 11th International Conference on Industrial and Information Systems (ICIIS).

[6] Shue S., Conrad J. A Survey of Robotic Applications in Wireless Sensor Networks. Proceedings of the IEEE Southeastcon 2013.

[7] Ryu J.H., Irfan M., Reyaz A. (2015). A review on sensor network issues and robotics. J. Sens..

[8] Ghosh P., Gasparri A., Jin J., Krishnamachari B. (2019). Robotic wireless sensor networks. Mission-Oriented Sensor Networks and Systems: Art and Science. Studies in Systems, Decision and Control.

[9] Luo L., Chakraborty N., Sycara K. (2015). Provably-Good Distributed Algorithm for Constrained Multi-Robot Task Assignment for Grouped Tasks. IEEE Trans. Robot..

[10] Dasgupta P., Jain L.C., Aidman E.V., Abeynayake C. (2011). Multi-robot task allocation for performing cooperative foraging tasks in an initially unknown environment. Innovations in Defence Support Systems-2: Socio-Technical Systems.

[11] Lim S., Rus D. Stochastic motion planning with path constraints and application to optimal agent, resource, and route planning. Proceedings of the 2012 IEEE International Conference on Robotics and Automation.

[12] Jones E.G., Dias M.B., Stentz A. (2011). Time-extended multi-robot coordination for domains with intra-path constraints. Auton. Robot..

[13] Lenagh W., Dasgupta P., Munoz-Melendez A. (2015). A spatial queuing based algorithm for multi-robot task allocation. Robotics.

[14] Gerkey B.P., Mataric M.J. (2004). A formal analysis and taxonomy of task allocation in multi-robot systems. Int. J. Robot. Res..

[15] Hojda M. (2015). Task allocation in robot systems with multi-modal capabilities. IFAC-PapersOnLine.

[16] Zitouni F., Harous S., Maamri R. (2020). A Distributed Approach to the Multi-Robot Task Allocation Problem Using the Consensus-Based Bundle Algorithm and Ant Colony System. IEEE Access.

[17] Comert C., Kulhandjian M., Gul O.M., Touazi A., Ellement C., Kantarci B., D’Amours C. Analysis of Augmentation Methods for RF Fingerprinting under Impaired Channels. Proceedings of the 2022 ACM Workshop on Wireless Security and Machine Learning (ACM WiSeML 2022).

[18] Gul O.M., Kulhandjian M., Kantarci B., Touazi A., Ellement C., D’Amours C. On the Impact of CDL and TDL Augmentation for RF Fingerprinting under Impaired Channels. Proceedings of the 48th Wireless World Research Forum (WWRF 2022).

[19] Gul O.M., Kulhandjian M., Kantarci B., Touazi A., Ellement C., D’Amours C. Fine-grained Augmentation for RF Fingerprinting under Impaired Channels. Proceedings of the IEEE 27th International Workshop on Computer Aided Modeling and Design of Communication Links and Networks (IEEE CAMAD 2022).

[20] Comert C., Gul O.M., Kulhandjian M., Touazi A., Ellement C., Kantarci B., D’Amours C., Traore I., Woungang I., Saad S. (2022). Secure Design of Cyber-Physical Systems at the Radio Frequency Level: Machine and Deep Learning-Driven Approaches, Challenges and Opportunities. Artificial Intelligence for Cyber-Physical Systems Hardening. Engineering Cyber-Physical Systems and Critical Infrastructures.

[21] Kuhn H.W. (1955). The hungarian method for the assignment problem. Nav. Res. Logist. Q..

[22] Nanjanath M., Gini M. Dynamic Task Allocation for Robots via Auctions. Proceedings of the 2006 IEEE International Conference on Robotics and Automation.

[23] Michael N., Zavlanos M.M., Kumar V., Pappas G.J. Distributed multi-robot task assignment and formation control. Proceedings of the IEEE International Conference on Robotics and Automation (ICRA).

[24] Viguria A., Howard A.M. (2009). An integrated approach for achieving multirobot task formations. IEEE/ASME Trans. Mechatron..

[25] Giordani S., Lujak M., Martinelli F. A Distributed Algorithm for the Multi-Robot Task Allocation Problem. Proceedings of the Trends in Applied Intelligent Systems 23rd International Conference on Industrial Engineering and Other Applications of Applied Intelligent Systems, IEA/AIE 2010.

[26] Trigui S., Koubaab A., Cheikhrouhoue O., Yousseff H., Bennaceurg H., Sritig M.F., Javed Y. (2014). A Distributed Market-Based Algorithm for the Multi-Robot Assignment Problem. Procedia Comput. Sci..

[27] Lukic M., Stojmenovic I. Energy-balanced matching and sequence dispatch of robots to events: Pairwise exchanges and sensor assisted robot coordination. Proceedings of the 10th IEEE 10th International Conference on Mobile Ad-Hoc and Sensor Systems.

[28] Luo L., Chakraborty N., Sycara K. Distributed algorithm design for multi-robot task assignment with deadlines for tasks. Proceedings of the 2013 IEEE International Conference on Robotics and Automation.

[29] Martin J.G., Frejo J.R.D., García R.A., Camacho E.F. (2021). Multi-robot task allocation problem with multiple nonlinear criteria using branch and bound and genetic algorithms. Intel. Serv. Robot..

[30] Shin H.S., Li T., Lee H.I., Tsourdos A. (2022). Sample greedy based task allocation for multiple robot systems. Swarm Intell..

[31] Li W., Delicato F.C., Zomaya A.Y. (2013). Adaptive energy-efficient scheduling for hierarchical wireless sensor networks. ACM Trans. Sensor Netw. (TOSN).

[32] AbdelSalam H.S., Olariu S. (2011). Toward efficient task management in wireless sensor networks. IEEE Trans. Comput..

[33] Edalat N., Chen-Khong T., Wendong X. (2012). An auction-based strategy for distributed task allocation in wireless sensor networks. Comput. Commun..

[34] Liu S., Qiu Q., Wu Q. Energy Aware Dynamic Voltage and Frequency Selection for Real-Time Systems with Energy Harvesting. Proceedings of the Design, Automation, and Test in Europe.

[35] Liu S., Wu Q., Qiu Q. An Adaptive Scheduling and Voltage Frequency Selection Algorithm for Real-time Energy Harvesting Systems. Proceedings of the 2009 46th ACM/IEEE Design Automation Conference.

[36] Moser C., Brunelli D., Thiele L., Benini L. Lazy Scheduling for Energy Harvesting Sensor Nodes. Proceedings of the IFIP Working Conference on Distributed and Parallel Embedded Systems.

[37] Moser C., Brunelli D., Thiele L., Benini L. (2007). Real-time scheduling for energy harvesting sensor nodes. Real-Time Syst. J..

[38] Zhu T., Mohaisen A., Ping Y., Towsley D. DEOS: Dynamic energy-oriented scheduling for sustainable wireless sensor networks. Proceedings of the 2012 Proceedings IEEE INFOCOM.

[39] La Porta T., Petrioli C., Spenza D. Sensor-mission assignment in wireless sensor networks with energy harvesting. Proceedings of the 2011 8th Annual IEEE Communications Society Conference on Sensor, Mesh and Ad Hoc Communications and Networks.

[40] Edalat N., Motani M. (2016). Energy-aware task allocation for energy harvesting sensor networks. J. Wirel. Com. Netw..

[41] Boyd S., Vandenberghe L. (2004). Convex Optimization.

[42] Ashraf N., Faizan M., Asif W., Qureshi H.K., Iqbal A., Lestas M. (2019). Energy management in harvesting enabled sensing nodes: Prediction and control. J. Netw. Comput. Appl..

